# Development and Application of Mucilage and Bioactive Compounds from Cactaceae to Formulate Novel and Sustainable Edible Films and Coatings to Preserve Fruits and Vegetables—A Review

**DOI:** 10.3390/foods13223613

**Published:** 2024-11-13

**Authors:** Viviane Priscila Barros de Medeiros, Kataryne Árabe Rimá de Oliveira, Talita Silveira Queiroga, Evandro Leite de Souza

**Affiliations:** Department of Nutrition, Health Sciences Center, Federal University of Paraíba, João Pessoa 58051-900, PB, Brazil; barrosviviane89@gmail.com (V.P.B.d.M.); kataryne.arabe@academico.ufpb.br (K.Á.R.d.O.); talitaasq@gmail.com (T.S.Q.)

**Keywords:** active packaging, polymers, quality parameters, vegetable matrices

## Abstract

The accelerated ripening and senescence of fruits and vegetables is characterized by various biochemical changes that hinder the maintenance of their postharvest quality. In this context, developing edible films and coatings formulated with natural and biodegradable materials emerges as a sustainable strategy for preserving the quality parameters of these products in replacement of conventional petroleum-based packaging. Recently, plant-based polymers, including mucilage from different cactus species and/or their bioactive compounds, have been investigated to develop edible films and coatings. As the available literature indicates, the *Opuntia* genus stands out as the most used for mucilage extraction, with the cladode being the most exploited part of the plant. Conventional extraction methods are widely employed to obtain mucilages, which are applied to fruits and vegetables after being combined with plasticizing and cross-linking agents. In general, these films and coatings have proven effective in prolonging the shelf life and maintaining the nutritional, physical, and sensory quality of fruits and vegetables. Given their preservation potential, combining cactus mucilages with bioactive compounds, probiotics, and prebiotics represents an emerging trend in developing functional films and coatings. However, some limitations have been identified, such as the underutilization of different species and parts of the plant, the lack of standardization in extraction methods, and the absence of studies on the effects of the physicochemical properties of mucilages in the formulation and characteristics of films and coatings. Therefore, overcoming these limitations is essential for developing edible films and coatings with enhanced techno-functional properties and greater commercial viability.

## 1. Introduction

Harvested products, especially fruits and vegetables, are metabolically active, undergoing ripening and senescence processes that must be controlled to prolong their postharvest quality [1]. These products have intrinsic properties and are exposed to extrinsic factors that accelerate postharvest losses. Intrinsic factors include physiology, size, and ripeness. In contrast, extrinsic factors include mechanical damage, microbial diseases (especially fungi), and the lack of or inadequate postharvest management [2]. The inefficient management of these processes can result in significant losses of nutritional and quality attributes and financial setbacks [1,3]. Postharvest losses in fruits and vegetables can be as high as 45% due to poor postharvest handling [4].

In this sense, maintaining the quality of fruits and vegetables becomes crucial for the agri-food sector. To improve the postharvest use of these products, the production of minimally processed fresh foods has increased [5]. Minimal fruit and vegetable processing involves applying operations such as washing, sanitizing, peeling, chopping, and partial preservation treatments. This allows for obtaining products ready for consumption with sensory and nutritional characteristics like fresh fruits [6]. However, processing can cause physical damage (e.g., a soft texture and flavor loss), enzymatic browning, increased ethylene production, and respiration for vegetables and fruits. In addition, vegetable and fruit cell rupture caused by cutting releases cellular contents and promotes microbial growth, reducing their potential economic value [7].

These changes in fresh and minimally processed products require conservation techniques. Cold storage, alone or in combination with other treatments, represents the most effective technique for maintaining the quality (physical, nutritional, and sensory properties) of fruits and vegetables, as it reduces the rate of many metabolic processes and controls microbial deterioration over time [8,9]. Although cold storage remains a fundamental postharvest strategy, its efficacy in preserving the optimum quality of fruits and vegetables during transportation and in markets has yet to diminish. Furthermore, some of these materials may suffer chilling injury at temperatures below 7 °C, which favors the development of fungal diseases [4]. These situations have directed integrating postharvest technologies, such as modified and controlled atmosphere packaging, to preserve horticultural products [10]. However, conventional food packaging materials like plastic are not environmentally friendly. For this reason, researchers have focused on developing innovative biodegradable packaging for application in the food industry, aiming to replace packaging made from petroleum derivatives [11]. Active packaging in the form of edible films and coatings could be a sustainable alternative to preserving the quality of fruits and vegetables that are fresh and minimally processed [6,12].

Edible coatings and films are semipermeable barriers that may positively influence vegetable tissue metabolism by protecting against mechanical, physical, and chemical damage (facilitating weight loss control, reducing breathing and perspiration processes, and preserving texture and nutritional properties), in addition to being able to carry antioxidants, antimicrobials, and other stabilizers, controlling microbial growth, and preserving fruit/vegetable quality for a more extended period [9,13]. Furthermore, edible coatings and films can be consumed with food, providing additional nutrients, improving sensory characteristics, and enhancing product quality [13,14].

The main components used for producing edible/biodegradable films and coating are biopolymers, such as carbohydrates–polysaccharides (chitosan, sodium alginate, gums, starch, pectin, and cellulose derivatives); proteins; a solubilizing medium (e.g., water and ethanol); and plasticizers. The advantages of using such components are their capability to provide moisture resistance, a water-soluble nature, gelling properties, and good thermal and mechanical properties [15]. Researchers have recently focused on using natural biopolymers from different plant-based materials, highlighting the mucilage derived from cacti, characterized as a subgroup of hydrocolloids that can form gels in the presence of water [16]. Cactus mucilages have already been investigated for creating films and coatings, demonstrating promising results for improving the shelf life of fruits and vegetables [1,6,9,16].

Cactus mucilage is a complex high-molecular-weight polymeric heteropolysaccharide that contains variable amounts of neutral sugars (D-galactose, D-xylose, l-rhamnose, and l-arabinose) and D-galacturonic acid [12]. These substances swell when mixed with water or form colloidal suspensions, producing a shiny coating on the surface of the fruit (or vegetable). In addition, these substances have hydrophilic properties that create a barrier against water evaporation from the plant tissue, slowing weight loss and increasing firmness [9]. Its application also reduces microbial growth by developing a modified atmosphere around the product and can facilitate the incorporation of additives, such as antimicrobials and antioxidants [12]. Cactus species are also rich in bioactive compounds, such as betalains, phytosterols, polyphenols, vitamins, and minerals, which can improve the nutritional composition of foods when incorporated into coatings applied to foods [17].

Mucilage and its bioactive compounds could be preferred to synthetic materials in edible packages due to their intrinsic properties, such as nontoxicity, biocompatibility, biodegradability, and adaptability. Mucilage also has other advantages, including producing low-cost films compared to most biopolymers and being obtained through an easy extraction process with a high yield [16,18]. However, it has disadvantages, such as low mechanical strength and a high affinity for water. The properties of cactus mucilage films and coatings are closely related to the composition and structure of the polysaccharide, which is highly branched and rich in hydrophilic groups [19].

This review updates the main production methods, chemical composition, and technological and functional properties of edible films and coatings formulated with mucilage and bioactive compounds from different cactus species. Furthermore, the effects of these edible films and coatings on the quality parameters of fruits and vegetables, as well as the market challenges and future perspectives, are presented and discussed.

## 2. Cactaceas: Genera, Species, and Plant Parts Used for Extraction of Mucilages and Formulation of Films and Coatings

Cacti are xerotrophic plants cultivated in Central and South America, Asia, and Southern Europe [20]. The family species have a high concentration of polysaccharides, especially mucilage [21]. Mucilage is a subgroup of hydrocolloids that can create gels in the presence of water [22]. *Opuntia* species are the most potential sources of mucilage among plants due to their abundance, biodegradability, and renewability. *Opuntia* mucilage is a complex material formed from carbohydrates, including L-arabinose, D-galactose, D-xylose, L-rhamnose, and D-galacturonic acid, in variable proportions [2]. In the *Opuntia* genotype, the cladode age, cultivation area, and pruning season affect its composition [13,19]. Furthermore, seasonal variations contribute to the physicochemical changes of mucilage, such as the relative amount of carbohydrates related to water availability to the plant or the effects of changes in pH and electrical conductivity [23].

According to the data shown in Table 1, the *Opuntia* genus is widely used to extract and formulate edible films and coatings applied to fruits and vegetables. The most employed species are *O. ficus-indica* [1,2,3,6,8,9,10,12,14,15,19,24,25,26,27,28,29], *Nopalea cochenillifera* [17,30], *O. dillenii* [31,32], *O. stricta L.* [4], *O. robusta* [21], *O. stenopetala* [13], and *O. oligacantha* [33]. The *Hylocereus undatus* species was also used to extract mucilage in a study [34]. *O. ficus-indica* has gained popularity as a potential source of additives for the food industry due to its nutritional properties, such as high calcium and potassium contents, a high water-retention capacity, and bioactive phytochemicals [12]. The mucilage in *O. ficus-indica* represents about 10% of the dry weight of cladodes and can retain more than 30% of the total water in the storage parenchyma. This anatomical characteristic contributes to its high drought resistance and remarkable water-retention capacity in the tissues, which is explained by the polymerization of sugars, the elasticity of the storage parenchyma cells, and the high water-retention capacity of the mucilage [26].

The mucilage of *O. ficus-indica* found in cladodes is a complex anionic polyelectrolyte polysaccharide formed by alternating galacturonic acid linked to rhamnose residues. Branches are galactose residues that carry sugars, such as xylose and arabinose, as substituents [5]. This compound can be an attractive natural edible coating with high nutritional value, besides helping to preserve fruits and vegetables [29]. In addition to the complex mixture of carbohydrates, it presents quantities of phenolic compounds (flavonoids and phenolic acids); vitamins (mainly ascorbic acid, vitamin B, and α-tocopherol); polyunsaturated fatty acids; and amino acids [15,27,29]. The hydrophilic characteristic of *O. ficus-indica* coatings could act as a barrier to water transfer, retarding transpiration and browning; maintaining fresh weight, firmness, and nutritional attributes; and controlling microbial growth, resulting in a more extended storage period for coated fruits and vegetables [27,28].

Although *O. ficus-indica* is the most studied species, other cactus species have also been characterized as sources of mucilage and other bioactive components. Among the other cactus species producing mucilage is the cactus pear (*Nopalea cochenillifera* Salm-Dyck), a plant with an acid crassulacean metabolism. This mechanism confers an efficient use of water, enabling the plant to thrive in arid and semiarid regions, becoming a vital forage resource for ruminant animals, especially during the dry seasons of the year [17]. Using edible coatings based on forage palm is a way to naturally incorporate antioxidants, such as soluble phenolics, into the treated product [17]. Belonging to the cactus family, *O. dillenii* is mainly distributed in tropical and subtropical regions, growing mainly in the arid or semi-desert areas of America and Mexico. *O. dillenii* polysaccharide is one of the main active compounds of this plant and is extracted mainly from the fruit and plant stem. The main constituents are arabinose, xylose, fructose, glucose, galacturonic acid, and rhamnose [31,32]. A previous study reported high aggregation in the mucilage obtained from *O. robusta*, which indicated that this species has a higher fiber content than other *Opuntia* species [21]. The species *O. stenopetala*, traditionally used for human consumption and livestock fodder, showed potential for use in the formulation of multilayer coatings [13]. Xoconostle (*O. oligacantha* CF Först), a traditional fruit from Central Mexico, contains phenolic compounds, flavonoids, and betalains, which confer antioxidant and antibacterial activities and can thus be ideally incorporated into edible coatings [33]. Pitahaya (*H. undatus*), commonly known as “dragon fruit”, is a native fruit from tropical regions of America that belongs to the climbing cacti family. The fruit has white or red flesh with numerous tiny black seeds. Edible films produced with mucilage extracted from dragon fruit peel, combined with glycerol and pectin, demonstrated improved mechanical properties [16]. The addition of pectin in cactus mucilage-based edible coatings is particularly associated with an increase in solid content in the film/coating-forming solution, enhancing the mechanical and barrier properties of the produced film/coating [2,13]. Glycerol, in turn, contributes to a more efficient interaction among the components in the film-forming solution, resulting in films with smooth, homogeneous, and compact microstructures [16]. Pitahaya films have strong potential to aid sustainable food production by reducing food waste (pitahaya peel), which can be used as coating and packaging to extend the shelf life of fruits and vegetables [16].

As shown in Table 1, cactus mucilage can be obtained from different plants and plant parts, including cladodes or pads [1,3,6,9,13,15], stems [3,4,35], and fruits [28,33]. The cladodes represent the most abundant plant portion in cactus cultivation areas and are widely used for the extraction of mucilage and its subsequent application as an edible coating [16,18]. Consisting of branches that exhibit variable morphology, cladodes can be globular, cylindrical, or flattened, and their dimensions differ according to the species, plant age, and the cultivation environment conditions [37]. These structures are covered by a waxy layer that reduces transpiration and are arranged to form a tree without a defined trunk and branches. The internal structure of the cladodes consists of a dense network of cells that contain substances capable of absorbing and retaining water, allowing the plant to survive in arid conditions. Additionally, the fine spines on the outer surface are essential to defend against animals [38].

Mucilage is stored in the cladodes in mucilaginous cells located in the chlorenchymatous and parenchymatous tissues, with the parenchymatous being the tissue with the highest mucilage content [20]. The mucilage extracted from the chlorenchyma, the outer layer of the cladodes, is characterized by a fibrous nature with an irregular structure and dimensions. For this reason, it is removed during the mucilage extraction process [17,20]. In contrast, the mucilage obtained from the parenchyma, which constitutes the inner portion of the cladodes, has a higher concentration of pectic substances. This characteristic enhances its effectiveness in formulating edible coatings for fruits and vegetables [6,8,17,20].

Regardless of the part used in extraction, in general, the life cycle of cactus mucilage encompasses the stages of biosynthesis by the plant, extraction and purification, storage, industrial applications, and its biodegradation in the ecosystem [1,4,9,16,39]. After extraction, the preservation of ideal physicochemical characteristics of the mucilage was observed for up to eight months of storage at low temperatures [39]. As for the biodegradability rate, mucilage-based films disintegrated after 35 days of soiling in organic compost [16]. However, it is noteworthy that, despite its importance, few studies address these aspects during their analyses, which is an essential fact for the valorization of the polymer as a sustainable strategy for food storage.

## 3. Main Extraction Methods and Physicochemical and Technological Properties of Cactus Mucilages

Cactus mucilages are complex polymeric molecules composed of branched polysaccharides that act as intricate molecular networks capable of absorbing water [21]. These natural colloidal structures possess technological, nutritional, and functional properties that can be industrially exploited [40,41]. In recent years, different methods for extracting cactus mucilages have been developed or optimized to increase mucilage yields and promote its use at the industrial level. As shown in Table 1, conventional methodologies, such as hot extractions, water immersion, or organic solvent use, remain predominant over more advanced methods, such as ultrasound-assisted extractions and microwave-assisted extractions. However, it was observed that conventional methods require more extended periods and high temperatures, which can favor the degradation of thermosensitive bioactive compounds, such as phenolic compounds and pigments present in cactus mucilage [42,43]. Although their low cost is an attractive feature of these methods, the excessive use of organic solvents can increase extraction costs and negatively impact the environment due to improper disposal [44]. In general, mucilages extracted by conventional and ultrasound-assisted methods show similar rheological behaviors; however, the ultrasound-assisted method results in a higher yield and shorter extraction time [45]. Although several extraction methods are used to obtain cactus mucilage applicable in coatings and edible films, no previous study has addressed the influence of extraction methods on the techno-functional attributes of these films and coatings [4,9,24,26].

The first step preceding mucilage extraction from cacti is washing the cladodes to remove dirt from the field, then sanitization with sodium hypochlorite [10,46]. After this process, the cladodes are peeled and manually cut into smaller fractions [4,10]. The next step involves immersing and homogenizing the cactus pulp in different organic solvent concentrations, such as ethanol [9], citric acid [30], or distilled water [15]. This process can be aided by equipment, such as magnetic stirrers, blenders, or food processors, and may occur under heating or at room temperature (Table 1). Thermal treatment is commonly applied during mucilage extraction to weaken molecular interactions between polysaccharide chains, facilitate water entry, and promote non-covalent intermolecular interactions with mucilage aggregates [47,48]. Next, the obtained mucilage is precipitated and washed with ethanol or isopropyl alcohol in varying concentrations (Table 1) to remove pigments and then directed to the separation stage [13]. Ethanol is the most used organic solvent for precipitating mucilages, while isopropanol is applied for extracting and purifying various biomolecules [47]. The separation stage of the mucilage can be carried out by multiple methods, such as filtration [6], decantation [10], evaporation [15], or centrifugation [10], adjusting the parameters of rotation, time, and temperature (Table 1).

After the separation stage, the wet mucilage is concentrated using convective drying [13] or freeze drying [31,32] at different temperatures and drying times. Conventional oven or tray drying is the most used method in the food industry; however, its low efficiency can cause physicochemical changes in the cacti using different times and temperatures [47]. As shown in Table 1, the temperatures used during the mucilage drying process range from 40 to 105 °C. The influence of drying using two different systems, i.e., convective drying and refractance window drying, on mucilages from *O. ficus-indica* and *Austrocylindropuntia cylindrical* dried at various temperature ranges (45–85 °C) was evaluated. The results show that increasing the temperature reduced the drying time in all experiments. Fourier Transform Infrared Spectroscopy (FTIR) analyses indicated that the mucilage and cellulose did not undergo significant structural changes due to the drying techniques employed [47]. However, the refractance window method reduced the mucilage drying time by half, emerging as a more efficient alternative. The final step for obtaining mucilage is grinding and sieving to achieve a fine powder. The final product of the mucilage extraction is a white, amorphous, and water-soluble material that can formulate films and coatings in liquid form [4] or powder form [49].

As characterized previously, due to their ease of execution, conventional methods using organic solvents or distilled water are the most employed for extracting mucilages from cacti to formulate edible films and coatings (Table 1). However, using these methods requires longer extraction times and high temperatures, which can result in the degradation of thermosensitive bioactive compounds. Although the cost of executing these methods is lower than that of non-conventional methods, the large-scale use of organic solvents can increase the process costs and present significant environmental challenges [42]. To mitigate the use of organic solvents during the mucilage extraction process, some studies performed mucilage extraction using only distilled water under different temperature conditions (25 and 90 °C) and volume proportions (in a general ratio of 1:1 *w*/*v*), with or without agitation (using a blender) [3,4]. Despite not being characterized, the mucilages obtained, when applied to fruits and vegetables, proved to be functional as a barrier against oxygen and ethylene, slowing down the rate of respiration and ripening [3,4].

Non-conventional extraction methods, such as ultrasound-assisted extractions [31] and microwave-assisted extractions [10,12,34], have also been employed for obtaining mucilages from cacti (Table 1). A microwave-assisted extraction uses microwave energy to heat the solvent and the plant simultaneously, promoting cell rupture and facilitating the release of bioactive compounds, such as mucilage. The main advantages of this technique include its speed, ease of execution, low cost, and reduced use of organic solvents. These factors make this method a sustainable and efficient alternative [18]. In turn, an ultrasound-assisted extraction is based on acoustic cavitation, generated by high-frequency ultrasonic waves applied to samples immersed in extracting liquids. This process causes the formation of microbubbles that, upon collapsing, release the energy responsible for breaking down cell walls and releasing bioactive compounds. In addition to increasing the efficiency of the extraction process, this technique also allows for a significant reduction in the volume of solvents used, making it a sustainable and economically viable alternative without compromising yields or process efficiency [42].

The yield of mucilage from cacti after extraction can vary depending on the part of the plant used, the extraction method, the solvents employed, and the pre-processing and purification steps [49]. In addition, other factors, such as the season, the plant genotype, the maturation stage, and the harvest time, can influence the yield and the physicochemical, technological, and functional properties of the cactus mucilage [23,38]. In particular, the stage of maturity and time of harvest can modify the yield, which is reduced with advancing maturity and the mucilage composition in terms of the concentration of carbohydrates, pigments (such as chlorophyll), and minerals (such as calcium) [22]. Young cladodes have a high content of water, phenolic compounds, and, mainly, soluble fibers, contributing to a higher mucilage yield. In contrast, in older cladodes (which face more significant climatic variations), an increase in the content of insoluble fibers and minerals is commonly observed [43]. However, galacturonic acid was identified as the main component in mature cladodes, being essential for film formation due to their viscosity properties, water retention, and ability to chelate Ca^2+^ ions [50].

Therefore, considering the biotic and abiotic factors involved in cultivating cactus species and selecting the appropriate extraction method is necessary to obtain mucilages from cacti with desirable physicochemical and technological characteristics. Mucilages of seven native cactus species from Brazil (*O. ficus-indica*, *O. cochenillifera*, *C. jamacaru*, *C. hildmannianus*, *P. gounellei*, *P. pachycladus*, and *T. inamoena*), obtained through aqueous extraction and precipitated with ethanol, showed high yields, ranging from 8.9% to 21.54%. The extraction process involved aqueous extraction followed by precipitation with ethanol. More specifically, the species *P. gounellei* (21.54%), *P. pachycladus* (20.80%), *O. ficus-indica* (19.05%), and *O. cochenillifera* (18%) exhibited the highest yields, suggesting the potential of these cacti as sources of mucilages applicable to the food industry [22].

The physicochemical properties of cactus mucilages determine the functionality and behavior of coatings during food storage and handling [51]. An early study analyzed the physicochemical, structural, and technological properties of mucilages from seven cactus species, including species native to the semiarid region of Brazil (*O. ficus-indica*, *Opuntia cochenillifera*, *Cereus jamacaru*, *Cereus hildmannianus*, *Pilosocereus gounellei*, *Pilosocereus pachycladus*, and *Tacinga inamoena*). The mucilages exhibited a light coloration, with luminosity (L) values ranging from 69.29 to 81.99 and low a* values, close to negative, indicating a tendency toward green [22]. The authors emphasize that the color of the mucilages can vary depending on the plant maturation stage, the amount of chlorophyll, the extraction and purification methods, and the solvents used in precipitation. Technologically, the mucilages demonstrated a high water-retention capacity, ranging from 2.40 to 23.01 g/g. In contrast, the oil-retention capacity was relatively low, between 2.14 and 3.02 g/g, which was attributed to the hydrophilic properties already known of mucilages. The mucilages exhibited satisfactory emulsifying, stabilizing, and foaming properties, highlighting their potential applications in food [22].

The particle size of mucilage can also influence the distribution of nutrients and their functional groups. Grinding and sieving techniques help obtain particles with a better water- and oil-retention capacity and optimize the release of bioactive compounds during processing [48]. Scanning electron microscopy (SEM) tests conducted on mucilages extracted from the parenchyma of *O. robusta* revealed aggregates of small particles with irregular shapes and fibrous dimensions more significant than the mucilage extracted from the chlorenchyma [48]. A study showed that reducing the particle size of the flour from the cladode of *O. ficus-indica*, obtained through grinding and sieving, altered the content of carbohydrates and lipids present in the mucilage [52]. Another study identified changes in the physicochemical and technological properties of mucilage powders obtained from two cultivars of *O. ficus-indica* and one cultivar of *O. robusta* (Robusta), collected during two distinct periods. The powders from mucilages harvested in February exhibited higher porosity, oil absorption, and retention capacity while showing a lower water-retention and swelling capacity. In contrast, the powders of mucilages collected in August exhibited smaller impermeable particles and superior hydrophobic properties and a better emulsifying capacity, stability, and viscosity [53].

The viscosity of these mucilages is influenced by the concentration of monovalent and divalent ions in their composition, and they may exhibit pseudoplastic behavior [39,54]. Due to their hydrophilic nature and the presence of calcium, cactus mucilages have a higher water-retention capacity compared to oil [23]. This characteristic allows for a more efficient interaction with water, promoting water retention and the homogeneous distribution of the mucilage during its application in films and coatings [55]. Edible films and coatings with homogeneous characteristics indicate a structural integrity associated with improved mechanical properties [56]. Several factors, such as the mucilage concentration, temperature, pH, porosity, and particle size, can influence the viscosity of cactus mucilages, potentially hindering their diffusion during the application of edible films and coatings on fruits and vegetables [23,52,54]. Thus, the viscosity of the mucilage should be adjusted to the application method, as less viscous solutions are preferable for spray applications. In contrast, more viscous solutions are suitable for other methods, such as immersion and spreading [57,58].

The barrier capacity of films against water molecules is associated with their efficacy in minimizing moisture loss or gain in foods or food products. Water vapor permeability (WVP) can be measured to evaluate moisture barrier properties [59]. This parameter reflects the film’s water barrier property between the environment and the food matrix, contributing to increased shelf life [23]. Films with low WVP are ideal, as they prevent moisture transfer and water loss from the food during storage, thus helping to maintain food quality [11,59]. However, due to their hydrophilic nature and chemical composition, edible coatings and films formulated with cactus mucilage generally exhibit high WVP, which may be related to the mucilage extraction method, type and concentration of the additive used, and the coating processing method [23,49,55]. To improve the techno-functional properties of coatings and films formulated with cactus mucilage, using additives to reduce the spaces within the polymer structure, thus hindering water vapor diffusion, has been a common approach [51,54,60].

The mucilage from the genus *Opuntia*, commonly used for formulating edible coatings and films, is a complex polymer with a branched structure composed of various sugar units. These sugars interact with the hydroxyl groups of uronic acids, imparting the mucilage with its technological and functional characteristics [31]. The spectra obtained through FTIR analyses are commonly used to evaluate the main functional groups present in cactus mucilages, relating the absorption frequencies obtained to known absorption frequencies of recognized chemical bonds [23]. The bands commonly identified are OH groups from polysaccharides, carboxylic acids, uronic acids, and α- and β-D-glucose configurations [23]. In addition to carbohydrates, cactus mucilage is an excellent source of proteins; minerals (calcium, copper, iron, zinc, and manganese); and various phenolic compounds that can act in preserving fruits and vegetables when applied in edible films and coatings [22,23].

## 4. Processing Methods and Characterization of Films and Coatings Formulated with Cactus Mucilages and Their Bioactive Compounds

Postharvest losses of fruits and vegetables represent a significant challenge for the agricultural sector. In recent years, edible packaging in films and coatings formulated with different biomaterials (carbohydrates, proteins, and lipids) has been widely used as an innovative strategy to minimize postharvest losses [46]. In general, the application of edible coatings helps maintain the firmness of the food, control the respiration rate, and regulate the ripening process, playing a crucial role in determining the shelf life, marketability, nutritional value, and functionality of horticultural products [17,29].

From this perspective, various polymers obtained from natural sources, such as starches, cellulose derivatives, pectin, carrageenan, chitosan, sodium alginate, proteins (of animal or plant origin), and plant-based mucilages have been explored for the formulation of edible films and coatings [10,13,60]. The biodegradability, biocompatibility, bioavailability, low processing cost, and high extraction efficiency, combined with their nutritional, physicochemical, and technological properties, make cactus mucilage a widely used raw material in the formulation of effective edible coatings and films aimed at extending the shelf life of various fruits and vegetables [3,4,17].

Although edible films and coatings based on cactus mucilage offer several advantages, they present some limitations compared to synthetic polymers, including a low yield in the extraction process [22], the use of organic solvents in conventional mucilage extractions [17,34], and variations in mucilage chemical compositions, which make standardizing coating formulations challenging [23]. Additionally, the high hydrophilicity of mucilage reduces the mechanical strength and barrier properties, resulting in films that are fragile and have a granular texture, necessitating the combination of mucilages with other compatible polymers and plasticizing agents to optimize their physical and functional properties [15,16,61]. These challenges highlight the need for research developments that advance current knowledge and provide insights to overcome these limitations, paving the way for more effective coatings suited to the food industry [39].

### 4.1. Techniques and Components Used to Formulate Films and Coatings with Cactus Mucilages and Their Bioactive Compounds

Table 2 summarizes the main methods and components used to develop edible coatings and films based on cactus mucilage and bioactive compounds to preserve fruits and vegetables. The main components used for formulating edible films and coatings include mucilage in powder or liquid form [4,6]; plasticizing agents (glycerol, Tween, or sorbitol) [15,17,24]; and cross-linking agents, such as calcium chloride [3]. Generally, the formulation process involves diluting and homogenizing the mucilage powder in distilled or deionized water in concentrations ranging from 0.004% (*w*/*v*) to 75% (*w*/*v*), followed by the addition of plasticizing agents, such as glycerol (0.1% up to 50% *v*/*v*), sorbitol (3.4% *v*/*v*), or Tween 20 (10% *v*/*v*), depending on the methodology used (Table 2). These solutions may or may not be subjected to heating and stirring to ensure a complete incorporation of the mucilage with the other components in the solution [34]. Thus, the method for developing coatings and films is simple and easy to execute.

The choice of plasticizer and its respective concentration is fundamental for obtaining coatings and films with satisfactory mechanical properties. As shown in Table 2, glycerol is the most used plasticizer for formulating edible films and coatings [4,17], followed by sorbitol [15] and Tween 20 [26]. Glycerol is widely used as a plasticizing agent in formulating edible films and coatings due to its structural characteristics, such as a low molecular weight, hydroxyl (OH) functional groups, and high compatibility with biological polymers [13,15,62]. These properties facilitate the diffusion of glycerol within the polymer matrix, promoting the breaking of intermolecular interactions to increase mobility and interactions among the compounds, contributing to a homogeneous distribution and maintenance of the film’s mechanical properties [62]. Furthermore, glycerol has hydrophilic characteristics, acting as a water-retention agent in the film-forming matrix during drying and storage processes [60]. A high solubility of the films is desirable, as this allows for rapid dissolution in the oral phase of digestion or easy removal during consumption [62].

Films formulated with mucilage extracted from *O. ficus-indica* cladodes show higher values of moisture, solubility, water vapor permeability (WVP), and elongation at break (EB). In contrast, films produced with *O. ficus-indica* mucilage and sorbitol show higher tensile strength [62]. The higher tensile strength of films with sorbitol may be associated with the higher molecular weight of this plasticizer, which increases intermolecular spacing, reduces mobility, and promotes cohesion among molecules, thus enhancing film strength [15]. Considering these aspects, glycerol-based plasticizing agents are widely applicable in edible-film formulations for products requiring greater flexibility, such as fruits and vegetables [5,8,15]. However, the glycerol concentration added to the coating solution should be considered, as an anti-plasticizing effect may occur when higher concentrations are used [16]. The increase in parameters, such as the thickness, elongation at break, moisture content, water solubility, and water vapor transmission rate (WVTR), is associated with a higher glycerol content [62]. Therefore, the selection of the plasticizing agent and the applied concentration should consider the desired properties of the film, as plasticizers like sorbitol, despite providing less elasticity, increase mechanical strength, a favorable attribute in formulations where mechanical resistance is essential [15].

Plasticizers in concentrations ranging from 10% to 30% (*w*/*w*) resulted in films firmly adhered to the plate, making removal difficult. On the other hand, films formulated with 50% plasticizers exhibited an excessively sticky texture. Films containing 40% (*w*/*w*) glycerol demonstrated good handling properties and an adequate appearance, making this concentration ideal for film development [61]. However, the concentrations of plasticizers added to coating solutions and edible films formulated with cactus mucilage vary substantially (Table 2).

Edible coatings are defined as a thin layer of semi-liquid material applied to the outer surface of foods, using techniques such as spraying, immersion, or brushing [36]. As presented in Table 2, the most used methods for applying mucilage-based coatings to fruits and vegetables include immersion, followed by an atomizing spray, a direct application multilayer system, brushing, sprinkling, and layer by layer. In contrast, the preparation of films involves the homogenization of mucilage with the other components of the film-forming solution and the dispersion of the solution in Petri dishes, which are then subjected to drying and directly applied to the fruit [15]. Due to its simplicity, immersion is the most widely used method for coating fruits and vegetables. This process is divided into three distinct steps: first, immersion, where the fruit or vegetable remains in contact with the coating solution for a specific time; deposition, which involves the adhesion of the solution to the food surface; and drying, corresponding to the evaporation of the solution, resulting in the formation of the coating adhered to the surface of the fruit or vegetable [3,8,9,30].

To achieve efficiency during the process, it is essential to consider the viscosity, density, and surface tension of the coating solution [57,58]. Fruits and vegetables with irregular shapes can hinder the uniform application of coatings, resulting in areas with inadequate coverage that promote unfavorable biochemical changes, compromising their shelf life. On the other hand, prolonged exposure to the solution can lead to excessive moisture absorption, while shorter contact times may result in an uneven coating [63]. During immersion in the coating solution, cross-linking agents can be used to enhance the adhesion of the coating to the food surface [49]. Cross-linking agents are widely used to improve the formulation of edible coatings intended to preserve fruits and vegetables [22,64]. Among the agents commonly employed for the formulation of coatings made with cactus mucilage are plant extracts [1,9], organic acids [12,28,49,64], and metal ions [9,65].

Calcium ion (Ca^2+^) is widely incorporated in CaCl_2_ solutions as a cross-linking agent, given its compatibility with anionic polymers [65]. This interaction promotes improvements in the techno-functional attributes of edible coatings, enhancing their stability and effectiveness in preserving fruits and vegetables [3,65]. When in contact with the citric acid present in the cell wall of fruits, Ca^2+^ prevents the alteration of the intracellular gel layer, helping to preserve the texture and firmness of the fruit. In addition, Ca^2+^ plays an essential role in regulating fruit ripening and senescence, contributing to its resistance to biological stress through the activation of secondary metabolism and stimulating the synthesis of bioactive compounds necessary for the nutritional quality of fruits [65,66]. The combined application of calcium chloride as a cross-linking agent in the cactus mucilage-based coating favored the creation of a modified atmosphere around the food’s surface (namely, tomatoes), which resulted in the preservation of the overall quality attributes and extended its shelf life for three weeks under room-storage conditions [3].

Other methods not applied in the reviewed articles have also been studied to prepare coatings, emphasizing electroplating. This is a technique in which metal ions are reduced to metal atoms using an electrolytic solution with the passage of an electric current and then deposited on the surface of another. Its ability to deposit various metals, alloys, and non-metallic materials (such as plastics and ceramics) without hazardous chemicals and with a lower environmental impact makes it extremely valuable. This technique has been used in several industries, such as food packaging [67].

The development of film and coating formulations involving the combination of cactus mucilage with other biopolymers, such as aloe vera gel [1,9], gelatin [15], and cassava starch [30], has been explored to preserve fruits and vegetables (Table 2). The mixture of mucilage, cassava starch, and glycerol was reported to result in a negative interaction with the plant tissue of minimally processed yam, as this combination was not effective in maintaining sensory quality during refrigeration storage, unlike yam coated only with mucilage and glycerol [30]. In contrast, the edible coating made with *O. ficus-indica* mucilage combined with gelatin, probiotic strains (*Enterococcus faecium*), and glycerol demonstrated efficacy in preserving freshly cut apple slices, extending their shelf life and maintaining quality attributes during storage [15].

In addition to these biopolymers, bioactive compounds have also been used in coating formulations based on cactus mucilage to enhance the functionality and antioxidant action of the coatings [12]. An early study reported that adding glycerol and L-glutamine to the coating improved the nutritional profile of loquat fruit during cold storage [10]. Similarly, adding glutathione to the polysaccharide-based coating of *O. dillenii* improved quality parameters and extended the shelf life of freshly cut Chinese cabbage by inhibiting physiological processes [32]. In another study, the coating enriched with ascorbic acid effectively preserved phytochemicals and antioxidant activity in pecan nuts [12]. Edible coatings and films formulated with cactus mucilage are strongly affected by the cactus species, cultivation conditions, extraction method, and the concentration of mucilage and other components used in the formulation [27,28,49]. For this reason, understanding these aspects and knowledge of cactus mucilage’s physicochemical and morphological properties is essential for formulating edible coatings and films with satisfactory technological attributes [49].

### 4.2. Characterization of Microstructural, Functional, and Thermal Properties of Films and Coatings Formulated with Cactus Mucilages and Their Bioactive Compounds

The microstructural, functional, and thermal properties of films and coatings formulated with cactus mucilages and their bioactive compounds are strongly associated with the mucilage’s chemical composition and structural characteristics [49]. Although their importance is recognized, studies investigating these parameters during the formulation of coatings applied to fruits and vegetables are still scarce. This gap hinders the integrated interpretation of the coating’s morphological, functional, and thermal characterization variables [12,16,32]. In contrast, most studies emphasize the effects of these coatings on the overall quality attributes of fruits and vegetables. Therefore, this section will also present information on developing cactus mucilage-based films and coatings not applied in food matrices to deepen knowledge.

The mucilage’s particle size significantly influences the film’s mechanical properties. When used alone, the *O. ficus-indica* mucilage, in its smaller fraction (NC-120), did not result in adequate film formation, leading to a fragile and brittle matrix [52]. To overcome this limitation, cassava starch was incorporated into the formulation, which resulted in a homogeneous, easy-to-handle film. In another study, *O. ficus-indica* mucilage used for film production exhibited an amorphous shape with a random distribution of irregular particles in the form of aggregates due to calcium and other polyvalent ions. Scanning electron microscopy (SEM) images of the films made with mucilage revealed a porous, network-like structure capable of storing particles, gases, ions, and moisture, which are essential characteristics for the coating functionality [49]. On the other hand, films formulated with *O. ficus-indica* mucilage, with the addition of chitosan (2% *v*/*v*) and glycerol (3% *v*/*v*), exhibited a homogeneous, non-porous surface, although with a slightly darker color [2]. Color is a crucial attribute to consider when making edible films, as it directly influences consumer acceptance and must be compatible with the food to which the film will be applied [16].

The thickness of the films is considered an important physical parameter, as it affects water vapor permeability and mechanical strength. This parameter is directly related to the volume of the film-forming solution applied and the concentration of the dry matter in the solution [11]. Additionally, increasing thickness can reduce film transparency and decrease light transmission, which is advantageous for protecting photosensitive constituents in fruits and vegetables [23]. Increasing the concentration of mucilage typically results in increased water vapor permeability, which can directly impact the film’s functionality. In general, carbohydrate-based films exhibit a weak water vapor barrier due to their structure and composition, which provide excellent affinity for water vapor molecules [49]. Barrier properties are essential in preventing oxidation and microbial spoilage, directly contributing to extending the food’s shelf life [19]. High water solubility, low tensile strength, and low water vapor permeability can limit the use of mucilage alone, making the incorporation of additives and other polymers necessary [23].

The gas permeability of edible films and coatings plays a crucial role as a barrier in gas exchanges, particularly for carbon dioxide (CO_2_) and oxygen (O_2_), which are critical factors in the deterioration of fresh fruits and vegetables [60,63]. Low O_2_ levels and high CO_2_ levels help control microbial and enzymatic activities, extending the shelf life of fresh fruits and vegetables. This modified atmosphere also reduces the transmission of ultraviolet (UV) light, which limits oxidative processes, preserves sensory attributes, and slows nutrient degradation, promoting product quality conservation [28,56,64]. Due to the polymeric structure of mucilage and its compact network of hydrogen bonds, cactus mucilage-based films and coatings exhibit good barrier properties against gas exchanges [20]. These coatings reduce oxygen permeability and limit carbon dioxide accumulation around fruits and vegetables, helping to preserve quality and minimize economic losses along the supply chain [19,20,68].

Glycerol is the most used plasticizing agent for formulating mucilage-based films, as it improves the mechanical and thermal properties of the film. However, its addition can also negatively affect the barrier properties. The effects of applying plasticizers, such as glycerol, sorbitol, and polyethylene glycol, on the properties of films formulated with mucilage extracted from *O. ficus-indica* cladodes were investigated. All plasticized films exhibited lightness; yellow-green coloration; and a homogeneous, smooth, tasteless, and odorless appearance. The films with plasticizers showed good extensibility but poor resistance regarding mechanical properties. Furthermore, the use of polyols did not affect the thickness and solubility of the films in water. However, it increased the moisture content and reduced thermal stability, with the highest thermal stability obtained in the film formulated with polyethylene glycol due to its lower hydroxyl-group content [61].

Mechanical properties, such as tensile strength and elongation at break, are important indicators of the performance of edible films. Tensile strength refers to the maximum force a film can withstand per unit area before breaking, while elongation at break measures the change in the film’s length in the longitudinal direction at the point of rupture [11]. The glycerol concentration added to the formulation must be considered, as high concentrations can decrease tensile strength and increase elongation at the break of the formulated films [16]. The harvest of *O. stricta* clones (Orelha de Elefante Mexicana) and *Nopalea cochenillifera* (L.) Salm-Dyck (Miúda), at 6 a.m., positively impacted the films’ mechanical properties, permeability, and thermal stability. Specifically, the films formulated with the Orelha de Elefante Mexicana clone, harvested during the rainy season and the transition between the rainy and dry seasons, showed a darker color, improved the mechanical properties and water permeability resistance, and had a more compact microstructure and higher thermal stability [23].

Another study identified mucilage and films developed from the cactus mucilage of *N. cochenillifera* (L.) Salm-Dyck (Miúda) and *Opuntia stricta* (Haw.) (Orelha de Elefante Mexicana), which exhibited physicochemical stability over eight months of storage. After this period, changes in the appearance of the films were identified [39]. The results indicate that both species were water-soluble; however, *N. cochenillifera* demonstrated superior water- and oil-retention capacities and a higher concentration of phenolic compounds, resulting in films with enhanced mechanical properties compared to those formulated with *O. stricta*. These findings highlight the potential of these species for industrial applications, especially in the formulation of edible films and coatings with extended preservation properties.

Film biodegradation is also frequently assessed under anaerobic conditions through weight loss analyses. This method evaluates the degradation of polymeric materials when buried in organic matter-rich soil, determining the time required for complete decomposition [49]. Biodegradation occurs through the fragmentation and mineralization of the material caused by external factors, such as heat, humidity, and the enzymatic activity of microorganisms present during composting [49]. Films formulated from pitaya began to degrade after 28 days, with complete disintegration occurring after 35 days [16]. Similar results were reported for films made from *O. ficus-indica* mucilage, which started biodegrading after 24 days, with complete degradation achieved after 40 days [49].

As highlighted in the studies above, cactus mucilage-based films and coatings stand out for their flexibility, gas barrier properties, thermal stability, and biodegradability. However, these composite materials have some limitations, such as low mechanical strength and a high affinity for water, which may compromise their performance. Therefore, developing studies to improve these aspects is essential to expand their applicability and efficacy.

## 5. Effects of Films and Coatings Formulated with Cactus Mucilages and Their Bioactive Compounds on the Postharvest Quality and Storability of Fruits and Vegetables

**Figure 1:**
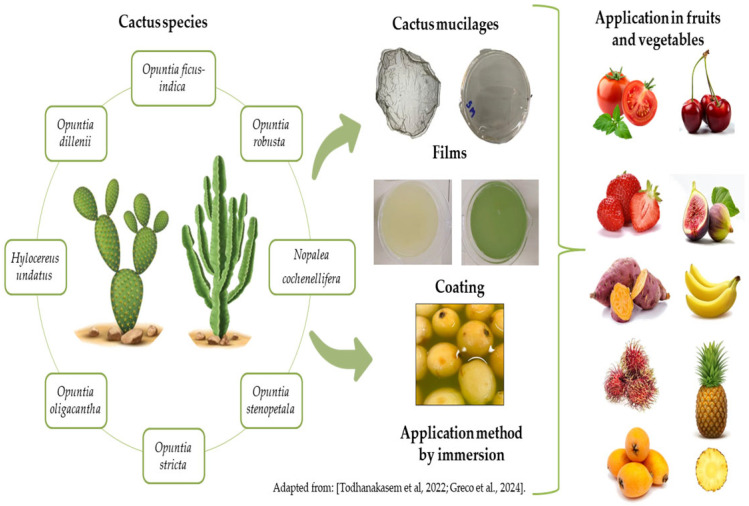
Applying cactus mucilage films and coatings to fresh and minimally processed fruits and vegetables [10,15].

Quality parameters, such as weight and firmness, color, soluble solid content, acidity, pH, ascorbic acid (vitamin C), and total phenolic content, change in fruits and vegetables (fresh and minimally processed) during storage, mainly as a result of physiological disorders (alterations in ethylene production, respiration, transpiration etc.) and infections caused by several pathogens [14,29]. Therefore, the impacts of applying coatings and edible films based on cactus mucilages and/or their bioactive compounds on the quality attributes of different fruits and vegetables, both fresh and minimally processed, have also been investigated (Figure 1), and the main results are summarized in Table 3.

### 5.1. Effects on Shelf Life, Color, Firmness, Weight Loss, Total Soluble Solids, pH, Acidity, Ascorbic Acid (Vitamin C), Total Phenolic Content, Antioxidant Activity, and Sensory Parameters

Weight loss is essential in determining the postharvest quality and shelf life of fruits and vegetables. Weight loss is usually caused by the continuous loss of dry matter through respiration and water-loss transpiration processes [15]. Notably, the internal water fluidity of fruits and vegetables increases during storage, which increases water loss and decreases quality [7]. Applying edible coatings and films minimizes water loss by creating a thin semi-permeable layer over the external surface as a barrier and maintaining high relative humidity [2,29,30]. The slowed weight loss and extended shelf life in fruits and vegetables coated with mucilage from cacti might be attributed to the improved water retention facilitated by the hydrophilic nature of this material [10].

Studies also suggest that adding active ingredients, such as mucilage, does not affect the coating barrier’s functionality [4,6,13]. On the contrary, the combination of a mucilage coating and film with different treatments, such as UV-C irradiation, chitosan, glycerol, glutamine, probiotics, and aloe gel, were effective in decreasing transpiration and the consequent decline in weight loss, maintaining freshness and prolonging the shelf life of products [9,10,13,15,20].

Texture or firmness is a critical quality attribute in fruits and vegetables. It may be modified during the postharvest period due to high metabolic activity and physical damage, such as the breakage of cell wall components and shocks during postharvest handling [14]. Studies indicate that physical barriers generated by cactus mucilage coatings effectively reduce pectin degradation, with reduced weight loss and firmness [32]. This effect could be attributed to the calcium content in cactus mucilage, which interacts with pectic acid in cell walls to form calcium pectate, thus maintaining the integrity of cell walls in fruit tissues [46].

Greater firmness of tissues preserved with mucilage was also attributed to greater concentrations of pectin and protopectin during storage, suggesting lower cell wall hydrolyzing enzyme activity [20]. Enzymes, such as pectin methylesterase (PME) and polygalacturonase, responsible for the solubilization of pectic substances in plant cells, require high levels of O_2_ and ethylene for their action, parameters that are reduced/modified with the application of coatings [20,33]. Studies have reported the potential of cactus mucilage to act as a gas barrier by decreasing O_2_ permeability and stimulating CO_2_ accumulation around coated fruits and vegetables during storage, significantly reducing the respiration rate [27,28]. Additionally, the incorporation of *Fuchsia microphylla* (flowering shrub) into mucilage coatings also had a positive effect on controlling water loss, thereby increasing fruits’ firmness and, consequently, physicochemical quality [13].

Color is an essential indicator of the shelf life of fruits and vegetables, being a factor that directly impacts consumer choice and acceptability and the marketing of these products [7,10,28]. During ripening, changes in the colors of fruits and vegetables are observed due to the degradation of the pigment chlorophyll and the biosynthesis/cumulation of carotenoids [3]. In fresh-cut fruits and vegetables, color changes are also caused by the enzymatic oxidation of carotenoids and polyphenols, forming brown pigments (quinones) [27]. The peroxidase (POD) and polyphenol oxidase (PPO) activities causing the oxidation of phenolic compounds are related to the browning and loss of quality of many foods [12]. The stability of pigments provided by applying edible coatings formulated with cactus mucilage occurs due to reduced gas transfers [12]. This reduces respiration and ethylene production rates, delaying ripening and its associated modifications in fruits’ color development [3]. In addition, the application of mucilage-based coatings could retard the oxidation reactions and enhance the brightness and overall visual quality of fruits [12,17,46]. However, decreased brightness was observed in cold storage, which is probably correlated with a decrease in specific pigments, such as the content of betalains [27].

Changes in the content of total soluble solids (TSSs) in fruits and vegetables are associated with the biosynthesis of polysaccharides and accumulation of sugars during the ripening, volatilization of soluble compounds, evaporation of water, and variations in storage conditions, such as temperature and humidity [13]. At advanced stages of ripening, the disassociation of some molecules and structural enzymes in soluble compounds also increases TSS levels [4]. The effect of cactus mucilage edible coatings limiting the increase in TSS could be attributed to the role of coatings acting as a semi-permeable barrier against O_2_, CO_2_, and ethylene, reducing respiration, transpiration, metabolic activity, and ripening, leading to a decrease in accumulated sugars and polysaccharides [27].

Due to the ripening process, organic acid content declines in fruits and vegetables as storage time progresses, reflected in a reduction in titratable acidity [1]. The decreasing trend of titratable acidity could be due to metabolic activities that lead to the biosynthesis of sugars from organic acids in the postharvest period [29,53]. However, coating applications, especially when combined with cold storage, can interfere with metabolic activities by reducing the use of organic acid during respiration, delaying the postharvest ripening process in fruits [3,29].

pH is also associated with acidity, and its modification may result from respiration and the increasing concentration of CO_2_ during storage [7]. The reduction in the concentration of organic acids, caused by the ripeness and water loss of fruits, also causes variations in this parameter [20,53]. However, a gradual increase in the pH of coated fruits has been reported, indicating less accelerated metabolic processes [33]. It was suggested that including a *F. microphylla* extract in the coatings could control water loss, thus delaying the maturity of fruits and changes in ripening parameters [13].

Ascorbic acid is an essential quality indicator for fruits and vegetables due to its sensitivity to oxidation during food processing and storage [4,27]. Usually, ascorbic acid content declines during storage, with the rate affected by the genotype, maturity stage, and storage conditions [5,34]. In minimally processed fruits, physical injuries can accelerate ascorbic acid degradation [46]. Low oxygen levels and low storage temperatures are other factors that can mitigate vitamin C degradation [28]. Applying cactus mucilage coatings effectively reduces losses in ascorbic acid content during storage, mainly by forming an oxygen barrier [46]. However, despite these effects, its preservation is compromised by its susceptibility to rapid oxidation, and a decrease in the content of this compound is evident in senescence [33]. Another fact related to this compound was that adding ascorbic acid to cactus mucilage also helps mitigate the loss of hardness, possibly due to cross-links forming a firmer structure that restricts moisture transfer. This effect was attributed to reduced cell wall degradation and cell collapse in the coated samples [12].

Phenolic compounds, such as anthocyanins and flavonols, are crucial in fruit quality, influencing its appearance, taste, and nutritional value [33]. Studies have shown that phenolic compounds act as non-enzymatic antioxidants, which scavenge free radicals and protect cells against oxidative injury in fruits and vegetables [19,46]. As an elicitor, the cactus mucilage could enhance antioxidant activity, scavenging reactive oxygen species, protecting cell membranes, and peroxidation, which could further extend the shelf life of fruits and vegetables and preserve their nutritional quality [10,29]. Moreover, Cactaceae species are abundant in bioactive compounds, such as betalains, phytosterols, polyphenols, vitamins, and minerals, which may also increase the nutritional composition of foods [17].

Malondialdehyde (MDA) quantification is another parameter for evaluating the degree of lipid peroxidation in fresh-cut product tissues and cell membranes. The antioxidant effects of the cactus mucilage coating were reflected in the elimination of free radicals generated by cells, preventing the oxidation of membrane lipids, reducing the formation of MDA, and effectively delaying enzymatic browning [7].

Sensory quality directly influences the consumption of fruits and vegetables and is a significant indicator for measuring their commercial value [32]. Due to the possible sensory impact that films and coatings can cause, this parameter was evaluated in some of the retrieved studies in this review. The mucilage coating and combined components, such as glutathione and glycerol, enhanced the sensory profile and preserved the overall appearance but did not interfere with or alter the natural taste of products, an essential characteristic concerning edible coatings [6,46]. Cactus mucilage coatings also exalt some critical parameters, such as firmness, aroma, sweetness, and taste, that are particularly appreciated by consumers [10,27,36]. These aspects can also be related to the changes the coatings cause in the fruit ripening process, emphasizing the content of phenolics, flavonoids, anthocyanins, and volatile compounds [7,17,20,30].

### 5.2. Effects on Microbiological Parameters

Fruits and vegetables are very exposed to microbial decay [19]. Although there is a high presence of carbohydrates and low acidity in cactus mucilage, studies have found that applying coatings based on this polysaccharide can reduce the microbial count [9,24,46]. A reduced oxygen supply and oxidative stress induced by the coating may be responsible for inhibiting the growth of fungi and yeasts, the primary fruit contaminants [12]. The activity of bioactive compounds present in Cactaceae can also increase microbial efficacy and scavenge free radicals, mainly by altering membrane permeability, damaging the cell wall, and thus blocking the growth and infection processes, mainly of fungi [29].

This antimicrobial activity can also be attributed to combined applications with other compounds, such as chitosan, glycerol, and aloe gel, which are known to prevent the microbial spoilage of fresh produce [9,46]. Thus, combining treatments could further enhance the microbiostatic effect of mucilage [32]. Furthermore, the addition of probiotics in cactus mucilage has also been shown to be an alternative method for controlling microorganisms in fruits, providing health benefits [15].

The studies mentioned previously presented the beneficial effects of applying coatings and edible films made from cactus mucilage and its bioactive compounds for maintaining the postharvest quality of fruits and vegetables, such as weight loss, firmness, color, and biochemical parameters. They minimized the microbial contamination of several fruits and vegetables. Their combination with other ingredients improved the quality and efficacy of cactus mucilage, and different applicational methods also extended their shelf life. It is essential to highlight that variations in the mucilage extraction procedures presented in Table 1, including factors such as time, temperature, type, and volume of solvent, can influence the physicochemical characteristics of mucilage, which play a crucial role in the techno-functional properties of films and coatings that act in the preservation of fruits and vegetables [15,16].

## 6. Trends, Limitations, and Market Challenges of the Use of Films and Coatings Formulated with Cactus Mucilages and Their Bioactive Compounds in the Food Industry

Cactus mucilage has been used in the formulation of coatings and films to extend the shelf life of fresh and minimally processed fruits and vegetables. The results are promising, with significant improvements in quality indicator parameters after its application alone or combined with other components [13,21,26]. Thus, applying coatings and films formulated with cactus mucilage can reduce waste and the amount of fruits and vegetables discarded throughout the production chain. Additionally, cactus mucilage, as a polymer matrix, is inexpensive and widely available in semiarid environments, favoring income generation through a circular economy approach with the sustainable use of natural resources [19,22,23]. By incorporating additives and other polymeric materials, it is possible to develop formulations that can be used as a viable alternative to traditionally applied methods [23].

In addition, cactus mucilage-based films and coatings can also serve as carriers for other bioactive substances, such as plant extracts with high concentrations of phenolic or probiotic microorganisms. This combination can enhance their functional properties, including antimicrobial activity against spoiling microorganisms and food contaminants, and benefit human health. A pioneering study on the incorporation of probiotics in cactus mucilage-based coatings showed promising results, highlighting the formation of thicker films that effectively preserved the quality attributes and shelf life of minimally processed apples [15]. However, a reduction in probiotic viability was observed during storage at 4 °C for 7 days, indicating the need to add bioactive compounds, such as phenolic compounds, to stimulate bacterial metabolism and promote greater viability of microorganisms during storage [15]. The incorporation of plant extracts and prebiotics in cactus mucilage-based coatings and films has proven effective in reducing weight loss and maintaining quality parameters such as pH, titratable acidity, total soluble solids, and antimicrobial and antioxidant activities in fruits and vegetables, thereby extending their shelf life [13,33].

More research is needed to identify biopolymer film-forming mechanisms for industrial use and optimize their physicochemical, morphological, mechanical tensile, barrier, and transparency properties. This information will support further studies, leading to better biofilm formulations for industrial applications [23]. Moreover, developing commercialization studies, such as evaluating new processes, safety, and toxicity, is essential for market applications [16].

Despite significant progress in the research on cactus mucilage, several limitations still need to be addressed. Studies often utilize a limited number of cactus species and need more crucial information regarding the genotype of the plants, maturation stage, and harvesting period for mucilage extraction. Additionally, the absence of standardization in extraction methods and the few available data on the physicochemical and technological characterization of the mucilages used in formulating films and coatings to be applied on fruits and vegetables hinder an integrated analysis of the variables influencing product quality and the success of their application. Therefore, it is recommended that future research focus on the bioprospecting of new cactus species, optimizing extraction processes, exploring the physicochemical characteristics of mucilages, and developing films and coatings that ensure effective incorporation into fruits and vegetables.

## 7. Conclusions

This review considered studies that tested the production methods, chemical composition, and technological and functional properties of edible films and coatings formulated with mucilage and bioactive compounds from different cactus species and their effects on the quality parameters of fruits and vegetables. Cactus mucilage presents a sustainable alternative to synthetic coatings. It is also a viable option for large-scale applications in the food industry, besides adding value to regional products represented by cactus species, which are currently used mainly as forage. The cactus species most used for obtaining mucilage are concentrated in the genus *Opuntia*, and the cladode is the most widely used part in the extraction process and subsequent application as an edible coating/film in fruits and vegetables. Cactus mucilage exhibits important physicochemical properties for formulating edible films and coatings, which can be used alone or combined with other polymers and bioactive compounds, often incorporated through immersion. The main findings from the analyzed studies show that the formulated edible films and coatings exhibit ideal mechanical properties for prolonging the shelf life of fruits and vegetables.

These properties are achieved through effective barriers against gases, moisture, and ultraviolet light; the efficient control of respiration rates; reduced senescence; and decreased water permeability. These factors contribute to the maintenance of firmness, improvement of appearance, preservation of nutritional composition, and enhancements in antioxidant and antimicrobial activities, resulting in an extended shelf life for fruits and vegetables. It is important to emphasize that the techno-functional properties of the coatings are closely linked to the physicochemical characteristics of the extracted mucilages, including their chemical composition, polymer structure, viscosity, particle size, and affinity with the substances added to the formulation. This relationship highlights the importance of carefully selecting mucilages to optimize and ensure a better performance of the developed films and coatings. Despite the promising results presented in this review, some scientific gaps still need to be addressed for a practical application of cactus mucilage in fruits and vegetables, such as the influence of genotypic variability and the harvest time of plant matrices on the techno-functional properties on the formulated films and coatings.

## Figures and Tables

**Table 1:** Species and plant parts and methods and conditions for extracting mucilages and bioactive compounds from cacti.

Species of Cacti	Plant Part	Extraction Method	Extraction Procedure	References
*O. ficus-indica*	Cladodes	Hot extraction	Scraping of cladodes; pressing; sieved to separate pure mucilage from slurry; extraction in water at 25%, 50%, and 75%, (*w*/*v*); and pasteurization at 70 °C for 45 min.	[1]
*O. stricta* L.	Stems	Aqueous extract	Hygienization; peeling and cutting; addition of distilled water (1:1 *w*/*v*); blended for 3 min; filtration through cloth; precipitation of the filtrate using 20% isopropyl alcohol in a 1:1 (*v*/*v*) ratio; centrifugation of the precipitate at 2.683× *g* for 10 min; and drying of the precipitate at 70 °C for 4 h.	[4]
*O. ficus-indica*	Cladodes	Hot extraction	Court of cladodes; crushing; homogenization in water in a 1:1.5 (*w*/*v*) ratio and at 20 °C; heating at 40 °C for 90 min; centrifugation at 1450× *g* for 20 min; heating of the supernatant until half of the initial volume; addition of ethanol (1:2 *v*/*v*); storage of the solution at 4 °C for 48 h; and elimination of the supernatant and use of pure mucilage.	[9,24,26]
*O. robusta*	Cladodes	Hot extraction	Removal of chlorenchymatous tissue and parenchymatous tissue; homogenization with ethanol or water in a 1:1 (*w*/*v*) ratio; agitation at 50 °C for 2 h; crushing; resting; filtering; drying at 50 °C; and milling and storage at 25 °C.	[21]
*O. ficus-indica*	Cladodes	Hot extraction	Hygienization; removal of the parenchyma; immersion in hot water for 30 s; homogenization with water for 5 min; and filtration.	[6]
*O. stenopetala*	Cladodes	Hot extraction	Homogenization with distilled water in a 1:2 (*w*/*v*) ratio; magnetic stirring at 60 °C for 90 min; centrifugation for 10 min at 246× *g*; addition of 96% ethanol in a 1:2 (*v*/*v*) ratio with the supernatant; centrifugation at 61× *g* for 10 min; drying of precipitate at 40 °C for 24 h; and grinding and storage.	[13]
*O. ficus-indica*	Cladodes	Hot extraction	Mucilage used as a gel: peeling and slicing of cladodes; homogenization in a blender; heating of the paste at 40 °C for 90 min; centrifugation at 10,000× *g* for 10 min; recovery of the supernatant; pasteurization at 77 °C for 1 min; and storage at 4 °C. Mucilage used as a polysaccharide: peeling and slicing of cladodes; homogenization in a blender; heating of the paste at 40 °C for 90 min; centrifugation at 10,000× *g* for 10 min; boiling of the supernatant at 100 °C to 50–60% of the initial concentration; centrifugation; precipitation with 96% ethanol (1:1); incubation at 1 °C for 2 h; centrifugation at 3600× *g* for 5 min; drying the pellet at 50 °C for 12 h; and grinding and sieving.	[20]
*N. cochenellifera*	Cladodes	Alcoholic extraction	Removal of the epidermis; slicing of the parenchyma; homogenization with 99.5% ethanol in a ratio of 2:3 m/v for 60 s; addition of ethanol to precipitate the mucilage; filtration of the mucilage in polypropylene fabric; washing (2×) with 99.5% ethanol for pigment removal; drying at 55 °C for 24 h; and grinding to obtain dry powder.	[17]
*O. ficus-indica*	Cladodes	Aqueous extraction	Peeling and cutting the cladodes; homogenization; dilution of the extract in water (2:1 *w*/*v*); and obtaining mucilage.	[3]
*O. ficus-indica*	Cladodes	Microwave-assisted extraction	Hygienization; cutting into cubes; microwave heating (900 W for 2 min); homogenization; centrifugation at 12,500 rpm for 15 min at 4 °C; discarding of insoluble fiber; and decanting and separation of soluble fiber.	[10]
*O. ficus-indica*	Cladodes	Microwave-assisted extraction	Hygienization; removal of thorns; removal of chlorenchyma; cutting into cubes; heating in microwave oven (900 W) for 3–5 min; homogenization; centrifugation of pulp at 8117× *g* for 15 min at 4 °C; and decantation of mucilage.	[14,24]
*O. ficus-indica*	Pear fruits	Aqueous extraction	Hygienization; cutting; immersion in water (5:1 *w*/*v*); overnight incubation at room temperature; filtration of the mixture; and concentration of mucilage by evaporation.	[28]
*N. cochenillifera*	Cladodes	Acid extraction	Hygienization; removal of glochids; cutting of cladodes into cubes; immersed in a solution of citric acid (5 mg/L) for 10 min; and mesh filtration.	[30]
*O. ficus-indica*	Cladodes	Aqueous extraction	Peeling and cutting of cladodes; immersion in distilled water (5:1 *w*/*v*); incubation overnight; filtration; and concentration of the solution by evaporation.	[2]
*O. oligacantha*	Fruit (xoconostle)	Ultrasound assisted extraction	Addition of orange essential oil to soy lecithin and aqueous extracts of 20% and 10% xoconostle; sonication of the mixture for 30 min with a 6 mm probe at an amplitude of 80% and a frequency of 20 kHz; distribution of the droplets via a dynamic laser light; scattering technique with an angle of 90 °C; and storing the nanoemulsion at 6 °C.	[33]
*H. undatus*	Dragon fruits	Alcoholic extraction	Cutting and peeling; removal of the seeds; removal of the pulp; precipitation of the mucilage with ethanol in a ratio of 2:3 (*v*/*v*) for 24 h at 4 °C; filtration; and drying in an oven at 40 °C for 24 h.	[34]
*Opuntia* spp.	Stems	Aqueous extraction	Homogenization of mucilage (20% *w*/*v*) in distilled water; centrifugation at 4500 rpm for 10 min; and pasteurization of the supernatant at 70 °C for 45 min to obtain pure mucilage.	[35]
*Opuntia* spp.	Cladodes	Hot extraction	Hygienization; removal of thorns; cutting; heating in three buffer solutions (pH 7, 4, and 9); repose for 5 h in pH 10; separation of the precipitate of the supernatant; resting of supernatant in a refrigerator overnight; separation of the precipitate; and drying in an oven at 105 °C for 12 h.	[36]
*O. ficus-indica*	Cladodes	Microwave-assisted extraction	Peeling; slicing; microwave heating at 800 W for 4 min; separation of insoluble fibers in gauze; centrifugation of the supernatant at 8000× *g* for 15 min at 4 °C; lyophilization for 72 h; and storage at 25 °C.	[12,29]
*O. ficus-indica*	Cladodes	Hot extraction	Hygienization; removal of parenchyma; trituration in distilled water in a ratio of 1:1.5 (*w*/*v*) at 20 °C; heating of the solution at 40 °C for 90 min; centrifugation at 1450× *g* for 20 min; heating of the supernatant to half of the initial volume; precipitation with ethanol in a 1:2 (*v*/*v*) ratio; solution stored at 4 °C for 48 h; discarding of the supernatant; and obtaining hydrated mucilage.	[8]
*Nopal cactos*	Cladodes	Hot extraction	Homogenization for 30 min with distilled water in a 1:1 (*w*/*v*) ratio; heating of the suspension at 90 °C for 30 min; centrifugation at 1500× *g* for 20 min; addition of 96% ethanol to the supernatant in a ratio of 2:1 (*v*/*v*); centrifugation at 1500× *g* for 20 min; drying of the mucilage for 24 h at 70 °C; and pulverization to obtain the mucilage.	[5]
*O. ficus-indica*	Pads	Aqueous extraction	Hygienization; removal of the peel and thorns; cutting the pulp; extraction in distilled water in a 1:2 *v*/*v* (pulp:distilled water) ratio; resting the solution for 24 h at room temperature; filtration through nylon cloth; centrifugation at 12,880× *g* at 4 °C for 30 min; precipitation with 95% *v*/*v* ethanol; and evaporation of ethanol in a water bath at 50 °C.	[15]
*O. dillenii*	Cladodes	Hot extraction	Peeling and slicing of cladodes; drying at 80 °C for 6 h; sifting to obtain a fine powder; extraction of fat (Soxhlet); dispersion in distilled water under agitation; obtaining a suspension at a concentration of 5% (*w*/*v*); extraction of the suspension in a water bath at 80 °C for 4 h; filtration; removal of proteins; concentration of filtrates; precipitation with three volumes of ethanol; filtration; and lyophilization.	[31]
*O. dillenii*	Cladodes	Hot extraction	Peeling and slicing of cladodes; homogenization with distilled water in a 1:1 *w*/*v* ratio; heating in a water bath at 75 °C for 6 h; filtration; removal of proteins from the filtrate; addition of three volumes of absolute ethanol to the precipitate; filtration; and lyophilization.	[32]

**Table 2:** Development and application of coatings and edible films formulated with cactus mucilage and its bioactive compounds for preserving fruits and vegetables.

Cacti	Coating/Film Material		Formulation	Application Method	Fruits/Vegetables	Reference
*O. ficus indica*	Coating	-Mucilage powder (6% *w*/*v*) -Glycerol (10% *v*/*v*) -Tween 20 (10% *v*/*v*)	Homogenization of pure mucilage extract in distilled water and glycerol or Tween 20.	Immersion	Minimally processed kiwifruit	[24]
*O. ficus indica*	Coating	-Liquid mucilage (6% *v*/*v*) -Glycerol (10% *w*/*v*)	Homogenization of pure mucilage extract with distilled water and glycerol.	Immersion	‘Dottato’ figs	[26]
*O. ficus indica*	Coating	-Mucilage powder (0%,25%, 50%, and 75% *w*/*v*) -Aloe gel (0%, 25%, 50%, and 75% *w*/*v*)	Hydration of the cactus mucilage with distilled water and aloe gel in different combinations.	Immersion	Mangoes	[1]
*O. stricta* L.	Coating	-Mucilage powder (1%, 2%, and 3% *w*/*v*) -Glycerol (2% *v*/*v*)	Homogenization of the mucilage in distilled water and addition of glycerol. Then, the solution was dissolved for 3 min and centrifuged at 2683× *g* for 10 min to use the supernatant.	Immersion	Peppers	[4]
*O. robusta*	Coating	-Mucilage powder (10% *w*/*v*) -Ethanol (50% *v*/*v*)	Addition of water or ethanol in parenchyma or chlorenchyma mucilage powder (to obtain a solution with 12% soluble solids).	Brushing	Tomatoes	[21]
*O. ficus-indica*	Coating	-Liquid mucilage (* Np)	Application of liquid mucilage.	Immersion	Minimally processed rambutans	[6]
* Np	Coating	-Mucilage powder (1% *w*/*v*)	Homogenization of mucilage in distilled water, followed or not by ultrasound treatment (40 kHz and 480 W) for 10 min.	Immersion	Minimally processed potatoes	[7]
*O. ficus-indica*	Coating	-Mucilage gel (25% and 50% *v*/*v*) -Polysaccharide of *O. ficus-indica* (1% *w*/*v*) -Glycerol (5% *v*/*v*)	Homogenization of the mucilage gel or polysaccharide in deionized water, followed by the addition of glycerol.	Immersion	Cherries	[20]
*N. cochenellifera*	Coating	-Mucilage powder (0.004% *w*/*v*) -Glycerol (30% *v*/*v*)	Homogenization of the mucilage in distilled water, followed by the addition of glycerol.	Immersion	Sweet potatoes	[17]
*O. stenopetala*	Coating	-Polysaccharides from *O. stenopetala* (0.1% *w*/*v*) -Sodium alginate (0.5% *v*/*v*) -Lactic acid (1% *v*/*v*) -Chitosan (0.5% *w*/*v*) -Extract of *F. microphylla* leaves (0.5 % *v*/*v*)	Preparation of different solutions: -Chitosan or alginate solution with added polysaccharides from *O. stenopetala* isolated or in combination with the extract of *F. microphylla* leaves; -Homogenization of the mucilage gel or polysaccharide in deionized water, followed by the addition of glycerol.	Multilayer system	Tomatoes	[13]
*N. cochenellifera*	Coating	-Mucilage powder (0.004% *w*/*v*) -Glycerol (30% *v*/*v*)	Homogenization of cactus mucilage powder in distilled water and glycerol.	Immersion	Sweet potatoes	[17]
*O. ficus-indica*	Coating	-Liquid mucilage (50% *v*/*v*) -Calcium chloride (6% *w*/*v*)	Dissolution of pure mucilage in distilled water; previous application with calcium chloride solution.	Immersion	Tomatoes	[3]
*O. ficus-indica*	Coating	-Liquid mucilage (60% and 67% *v*/*v*) -Glycerol (30% and 40% *v*/*v*) -Glutamine (3% *w*/*v*)	Homogenization of mucilage to glycerol and glutamine.	Immersion	Minimally processed white-flesh loquats	[10]
*O. ficus-indica*	Coating	-Liquid mucilage (* Np) -Ascorbic acid (5% *w*/*v*)	Homogenization of mucilage with ascorbic acid.	Atomizing spray	Strawberries	[28]
*O. ficus-indica*	Coating	-Liquid mucilage (* Np)	Application of liquid mucilage.	Atomizing spray	Minimally processed loquats	[14]
*O. ficus-indica*	Coating	-Liquid mucilage (* Np)	Application of liquid mucilage.	Atomizing spray	Minimally processed cactus pear fruits	[27]
*O. ficus-indica*	Coating	-Mucilage powder (3% *w*/*v*) -Ascorbic acid (0.03%, 0.009%, 0.015% *v*/*v*) -Glycerol (50% *v*/*v*)	Homogenization of mucilage with distilled water under magnetic stirring for 4 h at 30 °C. Mixture of varying concentrations of ascorbic acid and glycerol.	Immersion	Pecan nuts	[12]
*N. cochenilifera*	Coating	-Liquid mucilage -Cassava starch (3% *w*/*v*) -Glycerol (1% *v*/*v*)	Homogenization of mucilage in a previously heated cassava starch solution (70 °C), followed by the addition of glycerol.	Immersion	Minimally processed yams	[30]
*O. ficus-indica*	Film	-Mucilage powder (20% *w*/*v*) -Chitosan (2% *w*/*v*) -Glycerol (3% *v*/*v*)	Homogenization of mucilage in distilled water by vortexing, followed by the addition of chitosan and glycerol. Addition of the solution to a Petri dish for drying at room temperature.	Direct application	Tomatoes	[2]
*Opuntia* spp.	Coating	-Mucilage powder (20% *w*/*v*) -Glycerol (25% *v*/*v*)	Homogenization of mucilage in distilled water and addition of glycerol.	Immersion	Carica papaya	[35]
*O. oligacantha*	Coating	-Bioactive extract of *O. oligacantha* (10 and 20% *v*/*v*) -Mineral oil (70% *v*/*v*) -Soy lecithin	Homogenization of orange essential oil (continuous phase) with soy lecithin (surfactant), followed by the addition of the aqueous extract of *O. oligacantha* (dispersed phase). The mixture was sonicated at 30 °C.	Sprinkling	Tomatoes	[33]
*H. undatus*	Coating	-Mucilage powder white dragon fruits (3% *w*/*v*)	Homogenization of mucilage in distilled water for 30 s.	Immersion	Cherry tomatoes	[34]
*Opuntia* spp.	Coating	-Cactus polysaccharide (0.5%, 1%, and 2% *w*/*v*) -Acetic acid (1% *v*/*v*) -Ascorbic acid (2% *w*/*v*) -Glycerol (1.5% *v*/*v*) -Sunflower oil (0.025% *v*/*v*)	Homogenization of cactus polysaccharide with acetic acid, ascorbic acid, citric acid, glycerol, sunflower oil, and distilled water.	Immersion	Citrus fruits	[36]
*O. ficus-indica*	Coating	-Mucilage powder (6% *w*/*v*) -Glycerol (10% *v*/*v*)	Homogenization of mucilage in distilled water and addition of glycerol	Immersion	Sweet cherries	[8]
*O. ficus-indica*	Coating	-Mucilage powder (1%, 2%, and 3% *w*/*v*) -Glycerol (0.1% *v*/*v*)	Homogenization of mucilage in distilled water utilizing a magnetic stirrer at 5000 rpm at 30 °C for 4 h, followed by adding glycerol.	Immersion	Bananas	[29]
*O. ficus-indica*	Coating	-Mucilage powder (15% *w*/*v*) -Glycerol (10% *v*/*v*) -Aloe arborescens gel (2% *v*/*v*)	Homogenization of mucilage in distilled water, followed by the addition of glycerol and aloe arborescens gel.	Immersion	Figs	[9]
Nopal cactus	Coating	-Nopal mucilage (4% *w*/*v*) -Glycerol (0.5% *v*/*v*) -Chitosan solution (1.5% *w*/*v*)	Homogenization of mucilage in distilled water and addition of glycerol. Immersion of fruit in mucilage solution; placed in drying for 2 min; and immersion in chitosan solution and drying.	Layer by layer	Minimally processed pineapples	[5]
*O. ficus-indica*	Film	-Liquid mucilage (50% *w*/*v*) -Pig skin gelatin (2.9% *w*/*v*) -Glycerol (3.4% *w*/*v*) -Sorbitol (3.4% *w*/*v*) -Probiotic (10^8^ CFU/mL)	Homogenization of mucilage, gelatin, glycerol, or sorbitol on a magnetic stirrer at 70 °C for 10 min. The film-forming mixtures were dispersed in Petri dishes and dried at 45 °C for 24 h.	Direct application	Apples	[15]
*O. dillenii*	Coating	Mucilage powder (0.5% 1% and 1.5% *w*/*v*)	Homogenization of mucilage in distilled water.	Immersion	Potatoes	[31]
*O. dillenii*	Coating	-Mucilage powder (1% *w*/*v*) -Glutathione (0.4% *w*/*v*)	Homogenization of mucilage in distilled water, followed by the addition of glutathione.	Immersion	Chestnuts	[32]

* Np: data not provided.

**Table 3:** The main effects of applying coatings and edible films formulated with cactus mucilage or its bioactive compounds on extending the shelf life and preserving the nutritional, physicochemical, and sensory qualities of fruits and vegetables.

Composition of Coatings and Edible Films	Applied Fruits and Vegetables	Storage Conditions	Quality Parameters Evaluated	Main Effects	References
Aloe (*Aloe debrana*) gel and *O. fícus-indica* mucilage	Mango in natura	25 °C, Np RH, for 16 days.	Sensory evaluation, pH, total soluble solids (TSSs), titratable acidity (TA), and sugar-to-acid ratio.	The application of the aloe gel influenced the sensory attributes, TSS, TA, and the sugar-to-acid ratio, while the cactus mucilage primarily affected the color, appearance, and overall acceptance. Application of the Aloe gel and cactus mucilage (50 and 75%), in combination and alone, had a tendency to retard quality deterioration and maintained a good appearance of the mangoes.	[1]
*O. ficus-indica* mucilage extract mixed with glycerol	Papaya in natura	27 °C, 55–60% RH, for 6 weeks.	Weight loss, ascorbic acid content, pH, firmness, TSS and microbial qualities (total mesophilic microorganisms (TMM), total psychrotrophic microorganisms (TPMs), yeast, and mold.	Mucilage presented a protective effect to the firmness in coated samples. The *O. ficus-indica* mucilage with glycerol was more effective than only mucilage extract in extending the shelf life of fruits, reducing their ascorbic acid content, pH, and TSS when compared to the control in the storage period.	[35]
*O. ficus-indica* mucilage	Minimally processed kiwifruit	5 °C, 90% RH, for 12 days.	Firmness, weight loss, TSS, TA, sensory evaluation, package O_2_ and CO_2_ analysis, ascorbic acid, microbiological analysis (TMM and spoilage and/or pathogenic groups), and pectin analysis.	Mucilage edible coating presented positive effects on the physical maintenance, ascorbic acid, pectin content, visual quality, and flavor score of the storaged fruits. Samples coated with mucilage and Tween 20 presented an increase in microbial growth, mostly of yeasts. The coating without Tween 20 was more effective for quality parameters in kiwifruits.	[24]
*O. ficus-indica* mucilage	‘Breba’ fig	4 °C, for 14 days.	Firmness, weight loss, respiration rate, ethylene production, color, visual appearance score, sensory analysis, TSS, pH, TA, total phenolic compounds (TPCs), total carotenoids, and microbiological analysis (TMM and spoilage and/or pathogenic groups).	The application of the mucilage edible coating was effective in maintaining the fresh fruit’s brightness, weight, visual appearance score values, firmness, and total carotenoid content. The coated fruit revealed an expressively lower growth of *Enterobacteriaceae* than a control. The association of temperature and mucilage was effective in reducing water transpiration and retaining the mechanical and chemical properties of the fig through 10 days of storage.	[26]
*O. stricta* L. mucilage	Pepper in natura	25 °C, 65% RH, for 6 days.	Weight loss, TSS, and iron and ascorbic acid content.	The cactus mucilage coating reduced the weight loss; maintained the TSS, iron, and ascorbic acid; and extended the shelf life of the peppers.	[4]
*O. robusta* mucilage	Tomato in natura	20 °C, Np RH for 21 days.	Weight loss, texture measurement (firmness), and determination of lycopene.	The mucilage obtained from the parenchymatous tissue was more effective as an edible coating than the mucilage of the chlorenchymatous tissue. Tomatoes coated with mucilage revealed significantly higher firmness and reduced weight loss and lycopene content than the control.	[21]
*O. ficus-indica* mucilage	Minimally processed rambutam	5 °C for 10 days.	Weight loss, color, firmness, TSS, and sensorial evaluation.	The mucilage coating effectively reduced weight loss and preserved the fruit’s firmness during storage. Additionally, the coating helped retain the fruit’s color and overall appearance. Sensory evaluations indicated higher acceptability and consumption intentions for coated fruits. Overall, the cactus mucilage coating extended the shelf life and maintained the fruit’s quality	[6]
Chitosan and *O. stenopetala* with alginate and *F. microphylla* extract	Cherry tomato in natura	20 °C, Np RH, for 15 days.	Weight loss, pH, TA, TSS, firmness, color, and microbiological assays (aerobic mesophilic microorganisms, molds, and, yeasts).	The multilayer coating applied controlled weight loss and maintained the pH, TA, and TSS. Further, the coatings did not impact the visual appearance of the fruit. In addition, a visual evaluation of the peduncle scar showed that the coatings incorporating *F. microphylla* prevented *F. oxysporum* growth, prolonging the shelf life of the cherry tomatoes to 6 days.	[13]
Cactus polysaccharides (CPs) and ultrasound (US)	Minimally processed potato	4 °C, Np RH, for 8 days.	Microbiological assay, color, texture analysis, TSS, pH, cooking loss, water status and distribution, enzyme extractions and assays, TPC, malondialdehyde (MDA) content, membrane permeability, antioxidant capacity, catalase activity, and volatile organic compounds.	The CP-and-US combined treatment improved the bacteriostatic effect, browning inhibition effect, and antioxidant capacity in the vegetable. Furthermore, it reduced the mobility of water and the degree of membrane lipid peroxidation, maintaining the appearance quality, texture properties, and cell membrane integrity. The combination of CP and US improved the shelf life of potatoes.	[7]
*O. ficus-indica* mucilage and chitosan	Cherry in natura	1 °C, 90% RH, for 28 days.	Weight loss, TSS, TA, pH, peel color, moisture/dry matter, respiration, firmness, microbial decay, resistance to pedicel removal, extraction of phytochemicals, TPC, total flavonoid content (TFC), anthocyanins, and antioxidant capacity.	The application of the coating minimised weight loss and respiration rates, while enhancing the firmness of the fruits. Additionally, the coatings increased the antioxidant content, including phenolics, flavonoids, and anthocyanins, compared to the control group. The application of the edible coatings preserved the quality and prolonged the shelf life of cherries during storage.	[20]
Cactus pear (*Napolea cochenellifera* Salm Dick.) mucilage	Sweet potato	8 and 23 °C, Np RH, for 14 and 26 days, respectively, (simulated refrigerated transport and shelf conditions, respectively).	Visual appearance, weight loss, firmness, color, TSS, starch, antioxidant capacity (DPPH and FRAP), total phenolic compounds (TPCs), ascorbic acid, and total carotenoids.	The packed vegetables, with or without coating, maintained greater visual scores after transference to room conditions, exhibiting a lower weight loss for up to 26 days. Additionally, by visual analysis, the firmness, antioxidant activity, and phenolic and starch contents were stable in raw and cooked sweet potatoes packaged (coated or not) for up to 26 days.	[17]
Cactus mucilage and calcium chloride (CaCl_2_)	Tomato in natura	21 °C, 45% RH, for 20 days.	Weight loss, fruit decay, firmness, TA, TSS, color, and ripening index.	The combined coating significantly reduced weight loss and decay and retained firmness, TA, and TSS compared to untreated fruits during storage. Additionally, the coatings delayed color changes, indicating a slower ripening process. The combination of mucilage and CaCl_2_ effectively preserved the quality and extended the shelf life of tomatoes under ambient conditions.	[3]
*O. ficus-indica* mucilage with glycerol and l-glutamine	Minimally processed loquat	5 °C, Np RH, for 11 days.	TSS, TA, antioxidant activity, extractable juice, weight loss, color, TPC, antioxidant activity, sensory analysis, and visual scores.	The formulation of a mucilage edible coating enriched with 30% glycerol and 10% L-glutamine provided better postharvest resistance than only a mucilage coating, maintaining the TSS, TA, and extractable juice; reducing weight loss; and increasing antioxidant activity and the nutritional profile in treated fruits throughout the entire cold-storage period. The coating did not compromise the sensory and visual quality of loquat fruits after the storage period.	[10]
*O. ficus-indica* mucilage with ascorbic acid	Strawbery in natura	4 °C, 85% RH, for 12 days.	Weight loss, color; overall quality; firmness; TSS; TA; ascorbic acid content; sensory analysis; and microbiological analysis (TMM, TPM, *Pseudomonas*, *Enterobacteriaceae* family, yeasts, and molds).	The coated fruits showed a linear increase in weight loss during storage. The ascorbic acid content and TSS increased in coated strawberries, and the overall visual quality decreased, being affected by storage. However, visual quality and sensorial analyses verified higher scores in the coated samples at the end of the storage period. Furthermore, the mucilage coating did not negatively interfere with the natural taste of the strawberries. The coating applications were not able to prevent microbial growth, but their development decreased in coated fruits.	[27]
*O. ficus-indica* mucilage	Minimally processed cactus pear fruits	5 °C, Np RH, for 9 days.	Firmness; TSS; TA; color; weight loss; bioactive compounds and radical scavenging activity; and microbiological parameters (TMM, TPM, *Pseudomonas*, *Enterobacteriaceae*, and yeasts); sensorial analysis; and visual score.	Mucilage had a barrier effect on minimally processed fruit during cold storage, which was revealed by the lower weight loss, higher firmness, maintenance of TSS, ascorbic acid and betalain contents, sensorial qualities, visual scores, lower respiration rates, and significantly less microbiological growth of the coated samples compared to the control during the storage period.	[28]
*O. ficus-indica* mucilage	Minimally processed loquat fruit	5 °C, Np RH, for 13 days.	Firmness; SST, TA, color, extractable juice, ascorbic acid content, weight loss, and sensorial analysis.	Mucilage coating preserved the quality, nutraceutical value, and sensorial parameters and improved the postharvest life of minimally processed fruits. The treatment did not impede microbial growth, but considerably reduced its development in coated fruits.	[14]
*O. ficus-indica* mucilage and Calcium ascorbate	Cactus pear fruits	5 °C, Np RH, for 9 days.	TSS; TA; carbohydrate; color; weight loss; headspace gas composition; nutraceutical attributes; sensory analysis and visual score; and microbiological analyses (TMM, TPM, *Pseudomonas*, members of the *Enterobacteriaceae* family, *Listeria monocytogenes*, and yeasts).	The coating treatment maintained the quality parameters, nutritional value, and sensorial profiles and enhanced the postharvest life of fruits. The coating application restricted the development of bacteria and yeasts and did not negatively affect the natural taste of fruits during refrigerated storage.	[46]
*N. cochenillifera* mucilage	Minimally processed yam	5 °C, Np RH, for 10 days.	Fresh mass loss, visual assessment, and sensory analysis.	The coating reduced dehydration, maintained sensory quality, and increased the amount of phenolic compounds in the yam during the storage period.	[30]
*O. ficus-indica* mucilage enriched with ascorbic acid	Pecan nut	60 °C, Np RH, for 25 days.	Color, hardness, microbiological analyses (total mold and yeast), TPC, TFC, antioxidant activity, and enzyme activity assays.	The enriched coatings effectively preserved the color, hardness, and overall appearance of the pecan nuts throughout storage. Additionally, the coatings maintained greater levels of TPC and TFC, besides antioxidant activities. The coatings reduced the activities of polyphenol oxidase and peroxidase enzymes, which was associated with quality deterioration. Overall, the coating protected the quality and extended the shelf life of pecan nuts under storage conditions.	[12]
*O. ficus-indica* mucilage (OM), chitosan, and glycerol	Tomato in natura	25 °C, for room RH, for 30 days.	Color, texture, and firmness.	The coated tomatoes displayed a uniform color and firmness comparable to the fruits under initial storage conditions and did not show symptoms of a microbial disease. The film revealed a strong antifungal effect against *Rhizopus stolonifer* in vitro and in situ and improved the shelf life of tomatoes.	[2]
*O. oligacantha* fruit	Tomato in natura	6 °C, Np RH, for 21 days.	Weight loss, firmness, color, pH, TA, TSS, texture, bioactive compounds, and antioxidant activity.	The nanoemulsion coatings, which included orange essential oils and xoconostle, effectively reduced weight loss and maintained the firmness, color, pH, TA, and TSS of the tomatoes during storage. Additionally, the coatings enhanced the antioxidant activity and preserved the histological structure of the fruit pericarp, indicating slower maturation. The coating application was capable for extending the shelf life and maintaining fruit quality.	[33]
Dragon fruit mucilage coating and UV-C radiation	Cherry tomato	4 °C, 95% RH, for 21 days.	Color; weight loss; TPC; TFC; ascorbic acid; total soluble solids; TA; and microbial analyses (aerobic bacteria, coliform, yeast, and mold).	The application of a combination of UV-C and an edible coating provided a higher ascorbic acid content, antioxidant capacity, and antimicrobial effect. The hurdle treatment reduced weight loss, and despite slightly modifying the color, it maintained the fruit’s visual appearance and extended its shelf life during the storage period.	[34]
Polysaccharide extract of *Opuntia* spp.	Citrus fruit in natura	5 °C, 90% RH, for 35 days.	pH, TA, and sensory evaluation.	The maximum moisture content and pH value were observed in fruits coated with *Opuntia* spp. polysaccharides. The coating application increased the shelf life and retained the sensorial quality of the citrus fruits.	[36]
*O. ficus indica* mucilage	Banana in natura	25 °C, Np RH, for 12 days.	Weight loss, decay incidence, ethylene production, respiration rate, TSS, TA, ion leakage, MDA content, color, total chlorophyll, degrading cell wall enzyme activities, total carotenoid content, protopectin, and firmness.	Mucilage coating positively delayed the fruits’ ripening process. The coated fruits presented higher firmness, chlorophyll content, and TA values and a lower TSS content, ethylene production, respiration rate, MDA concentration, ion leakage, and protopectin content than uncoated fruits.	[29]
*O. ficus indica* mucilage	Sweet cherry in natura	2 °C, 92% RH, for 14 days.	Fresh weight, TSS, TA, firmness, color parameters nutraceutical, and sensory analysis.	The treated fruits showed better acceptability, and quality parameters, such color, compared to the control group. The mucilage coating considerably reduced weight loss during storage and pedicel browning, enhancing the visual appeal of the cherries. The mucilage effectively increased the storage quality and shelf life of the coated fruits.	[8]
*O. ficus-indica* mucilage and aloe gel	Figs, ‘San Giovanni’ and ‘Melanzana’ cultivars	4 °C, 85% RH, for 12 days.	Weight loss; TSS; TA; Maturation Index; color; firmness; microbiological analyses (TMMs, yeasts, and *Pseudomonas* counts); TPC; and visual appearance score.	The combined application of *O. ficus-indica* mucilage and aloe gel edible coatings improved the fruits’ visual appearance, maintained firmness, and reduced weight loss during storage. The coatings extended the shelf life of the figs by maintaining their quality and reducing microbial contamination.	[9]
*O. ficus-indica* mucilage and probiotic strain *Enterococcus faecium* FM11-2	Minimally processed apple	4 °C, Np RH, for 7 days.	Weight loss	The application of the edible film resulted in improved preservation and shelf life of minimally processed apples, minimizing weight loss and maintaining freshness during storage.	[15]
Nopal cactus mucilage, pullulan, and chitosan	Minimally processed pineapple	4 °C, Np RH, for 18 days.	Weight loss; firmness; color; TA; pH; ascorbic acid content; and microbiological (molds, yeasts, and total aerobic and psychotropic microorganisms) and sensorial analyses.	The coating application reduced weight loss and preserved the firmness of the pineapple cubes throughout storage. Additionally, the coatings helped in retaining the color and overall appearance. Microbiological analyses showed a reduction in microbial load, indicating enhanced safety and an extended shelf life.	[5]
*O. dillenii* polysaccharides	Minimally processed potato	5 °C, Np RH, for 5 days.	Weight loss, firmness, color, respiratory rate, microbiological analyses (total viable counts), and total sugar.	The application of different concentrations of *O. dillenii* polysaccharides considerably reduced weight loss and preserved firmness and retained the color and overall appearance of the potatoes. The polysaccharide-based coatings presented antibacterial and antioxidant activities, contributing to the quality and extended shelf life of the fresh-cut potatoes.	[31]
*O. dillenii* polysaccharides with glutathione	Chestnut	3 °C, Np RH, for 10 days.	Weight loss, firmness, color, respiration rate, TSS, and sensory evaluation.	The application of coatings significantly dropped the respiration rate and weight loss, maintained the firmness of and reduced the browning of the chestnuts during storage. The coatings’ application was capable of preserving quality and extending the product shelf life of chestnuts by at least 4 days.	[32]

Malondialdehyde (MDA); data not provided (Np); relative humidity (RH); titratable acidity (TA); total phenolic content (TPC); total soluble solids (TSSs); total mesophilic microorganisms (TMMs); total psychrotrophic microorganisms (TPMs); 2,2-diphenyl-1-picrylhydrazyl (DPPH); ferric-reducing antioxidant power (FRAP).

## Data Availability

No new data were created or analyzed in this study. Data sharing is not applicable to this article.
